# A Case of Anasarca and Ascites, Depending on the Pressure of a Tumour upon the Large Blood Vessels

**Published:** 1845-10

**Authors:** Laurence Turnbull

**Affiliations:** Resident Physician of Blockley Hospital, Philadelphia


*A case of Anasarca and Ascites, depending on the pressure of a Tumour upon the large Blood Vessels.* Reported by LAURENCE TURNBULL, M. D, Resident Physician of Blockley Hospital, Philadelphia.

Joseph Turner, negro, thirty-five years of age, entered the black Medical Wards of Blockley Hospital, May the 5th, 1845, labouring under anasarca and ascites; the dropsical effusion having taken place several months previous. He had been examined before he came in by a physician of one of our City Hospitals, who, from there being a small tumour over the upper portion of the sternum, with pain in the left arm, pronounced it to be a case of double subclavian aneurism. Upon an investigation and personal examination by Dr. Gerhard, one of the visiting physicians, his diagnosis was organic disease of the heart. There was great dulness on percussion, over the region of the heart, with slight alteration of its sounds, but no aneurismal thrill in the tumour. The usual remedies were resorted to for diminishing the momentum of the circulation, and to promote the activity of the various serous emunctories, by which the amount of fluid was diminished for a time; but toward the commencement of July it again increased, so as to prevent him from remaining in the recumbent position. The dyspnœa continued to increase rapidly, and he died on the 25th of July, at 5½ A. M.

*Post Mortem.*—A large amount of serous infiltration of the upper and lower extremities. Immediately under the soft parts, and over the sternum, lay a tumour, encephaloid in appearance, fibrous and scirrhous in its texture. The bone under it was softened and denuded of its periosteum, caused, probably, by a blow upon the bone of long standing.

*Thorax.*—On opening the thorax, we perceived that this tumour passed under the clavicles, over the top of, and beneath the sternum several inches, downwards, so far back, in fact, as to press upon the trachea and vessels generally of the parts. The jugular vein of the right side was distended with clotted blood, as well as the veins apparently of every part of the body. The heart was very slightly hypertrophied, and the lungs healthy, though the left was very small, not over one-third the natural size. Slight pleuritic attachments existed upon the left wall of the thorax. In the pericardium was found about eight ounces of fluid, of a urine colour; in the pleural cavities a considerable quantity of similar fluid.

Upon a division of the cavas and arteries, in removing the thoracic viscera *en masse*, blood spirted from several of the divided vessels, overflowing the table. Immediately under the aorta lay a tumour, as large as a goose's egg, of the same nature as those previously mentioned, which, in addition to the latter, must necessarily have seriously interfered with the lungs and action of the blood vessels, especially the more passive and sluggish channels—the veins.

*Abdomen.*—The peritoneum contained several pints of limpid serum. The liver was congested, and the acini hypertrophied, but otherwise natural. The spleen was small and indurated. The kidneys were unusually firm in their texture.

*Remarks.*—1st. From the post mortem appearances I would draw the conclusion, that the pressure upon the lung, trachea, and bronchial tubes, by the tumour, and also the effused fluid, was the cause of the intense dyspnœa.

2d. That the pressure of the tumour caused obstruction to the returning blood in the cavas, distending them and inducing transudation of the watery portion of the blood into the surrounding cellular tissue.

3d. The inordinate action of the heart arose, probably, from the difficulty with which the circulating fluid was forced through these obstructions,—the aorta, subclavian and other arteries, from their engagement within these masses, being weakened in their activity, and therefore rendered less able to contend with the increasing opposition to the circulation.

[Although in the above case the tumour appears to have proceeded from the sternum, a strong analogy may be observed between it and the interesting class of cases to which the attention of the profession has been called by M. Gintrac and others, under the title of *intra-thoracic tumours*, a brief notice of which will be found in our Record, extracted from the eighty-fifth number of the Medico-Chirurgical Review, (July, 1845.)—EDITOR.]

